# Potential Role of Gut Microbiota in Induction and Regulation of Innate Immune Memory

**DOI:** 10.3389/fimmu.2019.02441

**Published:** 2019-10-25

**Authors:** Shikha Negi, Deepjyoti Kumar Das, Susanta Pahari, Sajid Nadeem, Javed N. Agrewala

**Affiliations:** ^1^Immunology Laboratory, CSIR-Institute of Microbial Technology, Chandigarh, India; ^2^Gastroenterology Division, Washington University in St. Louis, St. Louis, MO, United States; ^3^Immunology Division, Texas Biomedical Research Institute, San Antonio, TX, United States; ^4^Department of Microbiology, School of Medicine, The University of Alabama at Birmingham, Birmingham, AL, United States; ^5^Center for Biomedical Engineering, Indian Institute of Technology-Ropar, Rupnagar, India

**Keywords:** gut microbiota, macrophages, monocytes, innate immunity, innate memory

## Abstract

The gut microbiota significantly regulates the development and function of the innate and adaptive immune system. The attribute of immunological memory has long been linked only with adaptive immunity. Recent evidence indicates that memory is also present in the innate immune cells such as monocytes/macrophages and natural killer cells. These cells exhibit pattern recognition receptors (PRRs) that recognize microbe- or pathogen-associated molecular patterns (MAMPs or PAMPs) expressed by the microbes. Interaction between PRRs and MAMPs is quite crucial since it triggers the sequence of signaling events and epigenetic rewiring that not only play a cardinal role in modulating the activation and function of the innate cells but also impart a sense of memory response. We discuss here how gut microbiota can influence the generation of innate memory and functional reprogramming of bone marrow progenitors that helps in protection against infections. This article will broaden our current perspective of association between the gut microbiome and innate memory. In the future, this knowledge may pave avenues for development and designing of novel immunotherapies and vaccination strategies.

## Introduction

The host immune system has the two major arms of protection. The first is innate immunity, which is characterized by non-specific and rapid response against the infectious agent. The second is adaptive, whose hallmark is specificity and memory (1). In the absence of adaptive immune response, innate immunity takes charge of mounting a successful defense response in many organisms (e.g., invertebrates, plants) including mammals (2). Innate immune memory is an emerging concept initially coined by Netea and colleagues that defines the rapid protective response of innate cells to heterologous infections (3–5). This is accompanied by the epigenetic reprogramming that modulates their gene expression and metabolic state and thereby affects the physiology and function of innate immune cells (6, 7). Notably, innate immune memory does not lead to permanent changes in the genome of cells such as mutations and rearrangement of genes, a characteristic of adaptive immune cells (T cells, B cells) (6). Recent findings showed the presence of previously encountered memories even in the non-immune cells of the host (8, 9).

The key players mediating the communication of host and microbes are the sensors, known as pattern recognition receptors (PRRs), expressed by innate immune cells such as dendritic cells (DCs), monocytes/macrophages, and natural killer (NK) cells (10–13). These PRRs recognize microbe- or pathogen-associated molecular patterns (MAMPs or PAMPs) (14–16) PRRs mainly include the families of toll-like receptors (TLRs), nucleotide-binding oligomerization domain (NOD)–like receptors (NLRs), C-type lectin receptors (CLRs), and RIG-I–like receptors (RLRs) (10, 11). The microbiota recognition via these PRRs may induce the memory response upon primary exposure (17, 18). This immunological memory in innate cells has been associated with non-specific vaccination effects. For instance, NK cells and monocytes derived from BCG-immunized individuals displayed a heightened immune response upon re-stimulation and heterologous infections (19, 20). Further, there is evidence of innate memory induction by polio and measles vaccines in humans (21). However, much remains to be studied in the context of gut microbiota–induced innate memory.

Gut microbiota has been established to be a crucial regulator of immune cell development and function (10, 22). The gut microbes and mammals have coevolved and cohabitated for millions of years and exhibit a high degree of mutualism (23). While the microbes get a habitat and nourishment from the host, these microbes return the favor by regulating various host physiological functions, including dietary digestion, and imparting protective immunity against pathogens (24, 25). Further, gut commensal–mediated competition for habitat site, nutrients, or secretion of antimicrobial peptides aids in the maintenance of homeostasis (26, 27). Additionally, signals derived from gut microbes are suggested to tune the immune cells for pro- and anti-inflammatory responses that may affect the susceptibility to diseases (22, 28). Likewise, germ-free mice have been shown to possess immune defects and impaired defense systems (29).

In a healthy state, the immune system reacts against the pathogenic microbes via activation of the inflammatory response, while being tolerant of beneficial microbiota (24, 30). For instance, bacterial phyla such as *Bifidobacteria* and *Lactobacillus* are considered beneficial and thus classified as “symbionts.” On the other hand, few species of *Escherichia coli* are viewed as opportunistic pathogens (pathobionts) (31, 32). Thus, the intestinal immune system requires a careful surveillance system to constantly monitor the flora communities in the lumen for maintaining the host defense. It is well-documented that T cell homeostasis and differentiation and their function are extensively modulated by the gut bacteria (33). For example, *Bacteroides fragilis* and segmented filamentous bacteria (SFB) have been reported to induce Tregs and Th17 cell differentiation, respectively, in the intestine, thus affecting the host response to infections (34, 35). It is still unclear how the gut microbial population, and its components, could reprogram the innate immune cells to exhibit memory responses.

**Figure 1:**
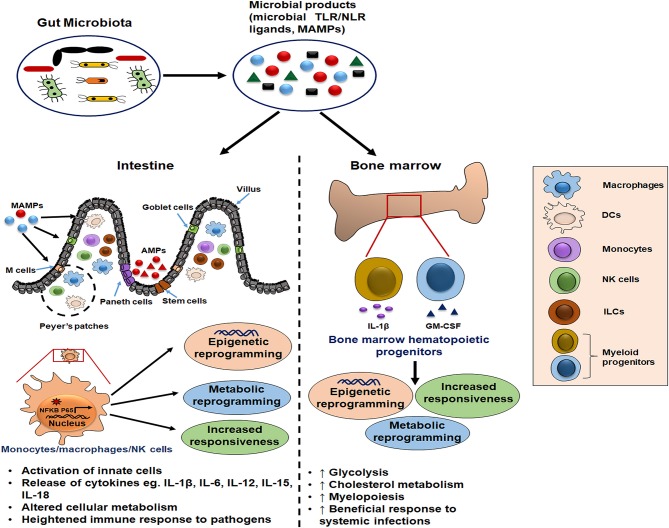
Schematic illustration of gut microbiota as potential inducer of innate memory. The gut microbial products serve as a source of microbe-associated molecular patterns (MAMPs) that bind pattern recognition receptors (PRRs) on innate cells such as monocytes/macrophages and natural killer (NK) cells. Further, this cell activation is accompanied by the epigenetic and metabolic reprogramming which is responsible for their increased cytokine release and heightened immune response upon the subsequent pathogenic exposure. Moreover, these microbial ligands reach the bone marrow through blood circulation and condition the hematopoietic progenitors to induce long-term memory traits and enhance myelopoiesis for mounting the beneficial inflammatory response during systemic infections.

Given the importance of gut microbiota, characterization and understanding of the involved microbial factors that determine the innate immune memory response is crucial for constructing novel therapeutic interventions (3, 7). This review provides current knowledge of gut microbial signatures and their interaction with the innate cells in imparting them the “memory” characteristics. It would be beneficial to develop immunotherapies and vaccination strategies that can generate memory features in innate cells to efficiently combat pathogens. Here, we discuss and hypothesize the possible impact of gut microbiota in inducing the beneficial innate memory response in the host (Figure 1).

## Prospective Link Between Gut Microbiota and Innate Immune Memory

**Figure 2:**
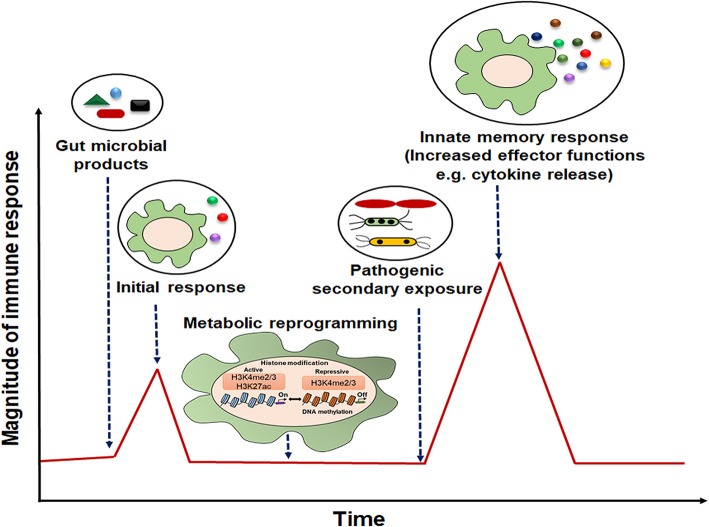
Representative model of innate immune memory response. After initial exposure to gut microbial components, innate cells with “memory” traits respond rapidly with high magnitude of immune response to the secondary stimulation.

The presence of microbiota-derived ligands/products/metabolites affects the differentiation and function of myeloid and lymphoid lineage innate cells via PRRs (36–38). Innate immune memory has been seen to be an attribute of myeloid cells (monocytes/macrophages), innate lymphoid cells (ILCs) including NK cells, and bone marrow progenitors (39). It is mediated by the transcriptional changes in genes or a specific locus and epigenetic rewiring of these cells upon the primary exposure (39). Consequently, the secondary response to the subsequent infections is enhanced, rapid, and nonspecific (Figure 2). This phenomenon also exists in the bone marrow progenitors, indicating the systemic effects of gut microbiota (40), and the induced memory may persist from weeks up to months (20, 41).

Training of PRRs expressing innate cells with gut microbial/non-microbial ligands is required as a protective mechanism independent of adaptive immunity during secondary infection/pathogenic exposures (42). For instance, administration of unmethylated CpG oligodeoxynucleotides prior to infection confers protection in a sepsis and meningitis model (43). Further, polysaccharide β-glucan has been reported to impart defense against *Staphylococcus* infection (44, 45). Other microbial components such as peptidoglycan that are expressed on numerous bacteria generate innate memory in *Toxoplasma* infection (46). In addition, cytokines such as IL-18, IL-12, IL-6, IL-23, IL-1β, and IL-15 have been demonstrated to generate memory response in innate cells (47, 48). Several studies established the existence of NK cell memory that leads to their increased activation upon second stimulation (43, 48). Moreover, DCs from protectively immunized mice demonstrated memory response against a fungal pathogen. These DCs showed increased IFN signaling pathway activation and specific histone (H3K4me3 and H3K27me3) modifications (49).

Importantly, commensals in the gut are involved in the production of immunomodulatory metabolites that comprise short-chain fatty acids (SCFAs) such as butyrate, acetate, and propionate (50–52). Further, commensals such as *Bacteroides, Lactobacillus*, and *Bifidobacteria* synthesize secondary bile acids that are derived from the metabolism of primary bile acids (53–55). Binding of these bioactive molecules to the receptors on the innate cells regulate their metabolism and functions (51, 56).

SCFAs serve as inhibitors of histone deacetylases (HDACs) in innate cells such as DCs and macrophages (57–59). Moreover, it was shown that SCFAs boost the population of myeloid precursors, resulting in protection against infection (60, 61). Additionally, secondary bile acids are known to modulate gut microbial composition (62). Further, they influence the function of innate cells by inhibiting NF-κB activation (63, 64). These findings suggest the possible epigenetic regulation of these cells via metabolites in the process of innate memory formation.

Emerging evidence suggests that diet-induced microbial changes in the gut could lead to the long-lasting rewiring of the innate cells. Interestingly, western diet (WD) has been linked to enhanced innate immune response. It is shown to induce epigenetic and transcriptional reprogramming of myeloid progenitors via the NLRP3 inflammasome and IL-1R signaling (65). In this case, WD-induced dysbalanced cholesterol biosynthesis led to the accumulation of mevalonate, which is implicated in the generation of innate memory. Indeed, a few studies indicate that maternal diet during pregnancy can have a pronounced effect in shaping the offspring microbiome (66, 67). Further, the gut may be a source of bacteria present in breast milk (68, 69). Of note, microbial exposure during pregnancy enhances the ILC3 and F4/80 (+) CD11c (+) mononuclear cell population in the gut of neonates along with the reprogramming of their intestinal transcriptional profiles. This effect is attributed to the transfer of maternal antibodies that retains the microbial signatures (70). In addition, infections during pregnancy can induce maternal immune activation (MIA), which can lead to the generation of immune cells with “memory” phenotype via epigenomic changes (71, 72). This can result in the condition of hyperimmune activation and neuropsychiatric diseases later in life.

Apart from the diet, stress is another emerging factor that can elicit innate memory phenotype (73, 74). Host cells secrete an array of small molecules upon stress or any cellular damage that can activate PRRs (75). These molecules, termed danger-associated molecular patterns (DAMPs), resemble PAMPs and thus are potential inducers of innate immune memory in cells (75). Social stress also releases IL-6, IL-1β, and TNF-α cytokines (73). Exposure to stressors has been demonstrated to trigger gut microbe–mediated release of MAMPs in blood circulation (76). Further, stressors implicate modification of H3K9 histones and the activation of transcription factor ATF7 (77).

Gut microbiota has been reported to play a key role in the induction of innate immune memory and protect against infections in both vertebrates and invertebrates (78, 79). These robust, non-specific memory effects mediated by MAMP–microbiota interaction may contribute to the development of effective vaccines and therapies that rely on boosting the host innate defense. Understanding the phenomenon and the involved mechanisms can be utilized to train these innate cells and enhance their function against pathogenic infections in the host. Further, this would aid the design and development of novel therapies to treat diseases (80).

## Mechanisms of Innate Memory Induction by Microbial Components

Recent studies have highlighted the array of mechanisms through which microbes imprint memory phenotype in innate cells. Transcriptional changes are the hallmark of memory imprints in innate cells, which involve chromatin modifications; specifically, the activation and expression of inflammatory genes take place many times higher than the basal level. This happens via the enhanced accessibility of DNA to enhancers/promoters, increased histone methylation, and acetylation along with enzyme RNA polymerase activity (81). These events are accompanied by transcription factor (NF-κB, STAT molecules, AP-1) translocation and activation (82–84). After the initial exposure to microbial ligands, DNA methylation/histone modifications continue to trigger rapid response upon re-exposure. Further, various immunological pathways such as STAT1, JNK, and MAPK are activated in the process of innate memory generation. For instance, MAPK activates the ATF7 transcription factor and decreases the repression of histones by recruiting the histone H3K9 dimethyltransferase (85). For instance, BCG changes the transcriptional signatures of hematopoietic stem cells (HSCs) to promote myelopoiesis and generate more potent macrophages that can protect against subsequent *Mycobacterium tuberculosis* infection (9). Of interest, a diet enriched in low-density-lipoprotein cholesterol such as WD elicits the expansion of HSCs along with the reprogramming of granulocyte monocyte precursor cells (GMPs) through the activation of NLRP3 inflammasome and possibly includes gut dysbiosis (65).

Importantly, cells with innate memory were reported to exhibit increased size, granularity, and activated phenotype (86). They are usually deprived of acetylation and lack active transcription. However, their inflammatory cytokine gene promoters are marked with histone methylation (H3K4), conferring them the attribute of rapid response upon re-exposure (87). These epigenetically rewired innate cells may be sustained in the host niche as seen in the case of NK cells and monocytes (40, 88). Interestingly, microbiota absence in mice impaired the histone modification in NK cells, rendering them unable to trigger a protective inflammatory response against viral infection (78).

Interestingly, various cellular metabolic pathways are involved in triggering and maintaining these epigenetic modifications (89, 90). Induction of memory features by β-glucan (microbial polysaccharide) is accompanied by a metabolic shift to aerobic glycolysis; this is referred to as the “Warburg effect” (91). Several studies reported that genes of the mTOR-HIF1α pathway were induced in β-glucan triggered monocytes that have undergone epigenetic changes, i.e., H3K4me3 and H3K27ac (7, 91). Moreover, TCA cycle metabolites such as mevalonate, succinate, and fumarate induce the activation of genes required to generate innate memory (92, 93). The pathway of cholesterol synthesis and mevalonate through the activation of IGF1 receptor and mTOR and further enrichment of histone H3K4me3 generated innate memory (93). There are also studies indicating the metabolic shift that leads to enhanced aerobic glycolysis, cholesterol synthesis, and NAD+/NADH ratio in cells (7, 91). Further, another metabolite, acetyl-CoA, has been shown to induce histone acetylation of genes related to glycolytic enzymes, such as phosphofructokinase, hexokinase 2, and lactate dehydrogenase (LDH), thus increasing glycolysis and inducing memory phenotype (94). In fact, β-glucan has also been shown to access bone marrow and act on myeloid-biased long-term HSCs (40). It is accompanied by changes in lipid metabolism, IL-1β signaling, and activation of the GM-CSF/CD131 axis. This β-glucan–mediated training is sufficient to protect against secondary challenges and recover from chemotherapy-induced myelosuppression.

The occurrence of innate immune memory relies on factors such as dose/amount and duration of initial inflammatory stimulus or PAMP. For example, a single low dose of lipopolysaccharide (LPS) was able to induce more release of proinflammatory molecules upon re-exposure and thus induce innate memory (86). In another study, a similar phenomenon was observed via epigenomic changes such as histone H3K4me1 modification. On the contrary, four-time administration of LPS led to the tolerant phenotype in cells (95). Moreover, Ifrim et al. reported that moderate to high doses of PRR ligands such as flagellin (10 μg/ml), LPS (100 μg/ml), and poly I:C (100 μg/ml) led to tolerance (86). On the contrary, a low to moderate dose of β-glucan (1 μg/ml) and muramyl dipeptide (MDP) (10 μg/ml) was able to elicit memory generation.

## PRR Mediated Regulation of Innate Memory by Gut Microbiota

Gut microbiota is a source of ligands that serve as MAMPs and activate innate cells expressing PRRs. In the homeostatic condition, the aberrant PRR activation is limited by various mechanisms such as the mucus layer (96); secretion of antimicrobial peptides, e.g., defensins (97); regenerating islet-derived protein 3 gamma (RegIIIγ) release by Paneth cells (98); secretory IgA (99); and inhibitory TLR signaling (100–102). Further, the PRR expression is context-dependent and varies in cell types to control the deleterious inflammatory response (16, 103). Interestingly, it is seen that the administration of *Lactobacillus plantarum* protected well against viral infections (104, 105). Further, there is emerging evidence that gut microbiota is known to affect innate memory phenotype at the distant mucosal sites or peripheral tissues. Yao et al. reported the immunological memory phenotype and protective functions in alveolar macrophages after respiratory virus infection (106).

In the context of memory, initial stimulation of innate cells by MAMPs serves as a factor for “priming” and functionally reprogramming of these cells to mediate heightened non-specific response to subsequent pathogenic exposure (39). Gut microbial components, mainly peptidoglycan, flagellin, β-glucan, and lipoproteins, may induce memory phenotype in the innate cells, which could underline their potential as an effective adjuvant for vaccination studies (107, 108).

Peptidoglycan: It is an important component of the bacterial cell membrane envelope, not present in the eukaryotic host. It is found in both gram-positive and gram-negative commensals (109). Further, the synthesis of peptidoglycan is ubiquitous in gut bacteria that is recognized by NOD receptors (110). For instance, NOD-1 binds only γ-d-glutamyl-meso-diaminopimelic acid (DAP)–containing muropeptides, whereas NOD-2 recognizes the MDP component (34). Further, a report demonstrated that NOD-2 induced H3K4me3 epigenetic modification in monocytes, a feature linked to innate memory (111). Moreover, NOD receptor activation triggers the inflammasomes to secrete cytokines such as IL-1β and IL-18, which is implicated in memory generation (112).

Flagellin: It is the essential component of many commensals and pathogens that activates TLR-5 signaling on innate cells (113). CD103+ DCs in the intestine recognize flagellin and secrete IL-23, which in turn triggers the ILCs to secrete IL-22 and thus facilitates innate defense (114).

β-glucan: This cross-linked glucan particle is commonly found in fungi and some bacterial cell walls (115). It is reported to induce long-term memory response in macrophages and bone marrow progenitors (40). This is accompanied by accumulation of metabolite mevalonate and Warburg effect in innate cells (91, 93). β-glucan is known to bind dectin-1 receptor (44, 116).

LPS: It is a glycolipid majorly found in the outer membrane of gram-negative bacteria and induces activation of TLR-4 and downstream NF-κB signaling (117). Earlier studies have shown that pretreatment with LPS prevents subsequent infection (118, 119). Further, LPS derived from gut microbes such as *Bacteriodetes* species is a potent activator of innate response (120), although the dose of LPS is a crucial factor to determine the induction of either memory or tolerant phenotype in cells (86).

Amongst the PRRs, TLRs are the most extensively studied transmembrane or intracellular glycoproteins that recognize a variety of microbial ligands or MAMPs as discussed above (10, 11). TLRs trigger signaling pathways, which leads to the secretion of cytokines and gene transcription in monocytes/macrophages and bone marrow progenitors (10). Triggering of TLR-2 by microbial lipoteichoic acids, lipoproteins, lipopeptides, and glycolipids activates NF-κB and is known to impart protection in intestinal acute inflammation (121). Additionally, initial priming of macrophages with TLR-2 and NOD-2 has been shown to confer protection against acute infection (105). This rapid response to continuously exposed mucosal tissue appears to be an essential part of host defense. In addition, a study demonstrated that stimulation of TLR-3 on macrophages with dsRNA could lessen the symptoms of DSS-induced colitis (122). Another study demonstrated the protective response in colitis upon TLR-3 activation by dsRNA of lactic acid–producing commensals (123).

In a healthy intestine, sensing of microbial LPS by TLR-4 is required to defend against invading pathogens (14). At homeostasis, there is a low level of TLR-4 expression, which gets elevated in inflammatory conditions and diseases. This results in the activation of innate immunity to restrict pathogenic exposure (124). Moreover, TLR-5 binds the flagellated bacteria and imparts protection to *Enterobacter* and *Salmonella* infections in the host (125, 126). Furthermore, another intracellular receptor, TLR-9, binds to the unmethylated CpG dinucleotides, which are abundant in commensals (127). TLR-9 activation stimulates the secretion of many proinflammatory cytokines including IL-12, which is considered a crucial cytokine to induce innate memory phenotype. Notably, it is the pathogenic microbe, not commensals, that breaches the gut lining and activate the basolateral TLR-9 receptor (128, 129). Notably, host genetic variations in the microbiota composition and pathogenic exposure could impact the commensal-mediated immune memory induction.

Another important class of PRRs involved in the education of innate cells is intracellular NLRs (130). Interestingly, commensal recognition by NLRs maintains homeostasis in the gut, while the pathogenic species of *Salmonella* and *Helicobacter pylori* trigger the inflammatory response (131, 132). NOD-1 and NOD-2 expressed in monocytes/macrophages are known to recognize microbial peptidoglycans DAP and MDP, respectively (34). NLR activation initiates the signaling pathways such as p38, MAPK, and NF-κB and elicits the release of cytokines (such as IL-1β and IL-18) that are known to induce innate memory phenotype (47, 48, 133). A study revealed that gut microbiota–derived peptidoglycan enhanced the pathogen-clearing capacity of bone marrow–derived neutrophils. The MDP fragments from the gut translocated to the bone marrow and triggered the neutrophil activation via NOD-1 signaling (134). This raises the possible hypothesis that gut microbial components reach peripheral sites and affect their epigenetic programming to imprint the memory phenotype (40).

Noticeably, these PRRs' stimulation may also lead to the tolerogenic phenotype, but this relies on the nature and duration of exposure to the initial microbial stimuli (135). Thus, these PRRs mediated modulation of innate cells should be monitored and utilized to generate the protective memory phenotype and ultimately the rapid, heightened, and efficient response against invading pathogens.

## Gut Microbiota Influences Functional Rewiring of Bone Marrow Progenitors

The impact of microbiota in the immediate sites of colonization such as the intestine, skin epithelium, and respiratory mucosa is quite plausible (27). However, its role in the primary site of hematopoiesis (bone marrow) may have significant immunological relevance. Long-term innate immune memory can be apparent in either the persistence of reprogrammed monocytes/macrophages in different tissues or modulation of bone marrow progenitors. This can be easily hypothesized, from the fact that LPS treatment in germ-free mice led to an elevation in the level of inflammatory cytokines and neutrophil recruitment and thus imparted systemic immunity (118). Concordantly, microbiota-derived peptidoglycan trigger NOD-1 receptor in peripheral blood neutrophils and boosted their anti-bacterial activity (134).

Monocytes, macrophages, and DCs that play a crucial role in shaping the immune response fall under myeloid-derived cells. Myeloid-derived cells originate in the bone marrow and then populate all lymphoid and non-lymphoid tissues. The myeloid cellular system has a non-redundant capacity to act in concert during the elimination process of pathogens and re-establishing tissue integrity (136). Recent reports demonstrated that gut bacteria, especially gram-negative bacteria, regulate granulopoietic events (137–140). Goris et al. showed that germ-free and polymyxin-treated mice have a lower number of bone marrow progenitor cells (141). Further, these germ-free mice complemented with fecal matter from wild type showed the reversion of the myelopoiegenic capability of precursors in generating colony-forming unit–granulocyte/macrophage (CFU–GM) colonies (142).

Interestingly, naïve mice with bone marrow transferred from the SFB and *Clostridium* spp. colonized mice demonstrated protection from *Entamoeba histolytica* infection. This is due to the expansion of marrow GMPs and increased expression of the epigenetic mediator JMJD3 in GMPs. SFB also altered the bone marrow DCs such that they have an enhanced capacity to secrete IL-23 (143). Further, adoptive transfer of DCs from SFB-supplemented mice to SFB-deficient mice was sufficient to protect against *E. histolytica* infection (143). Additionally, IL-1β and GM-CSF cytokines released from peripheral sites have been shown to confer the innate memory trait to the bone marrow cells (40, 65, 144).

The F4/80^hi^ macrophages have an embryonic origin, while F4/80^lo^ leukocytes have a hematopoietic origin (145, 146). To test the contribution of gut microbiota in the promotion of myelopoiesis, germ-free and specific pathogen–free (SPF) mice were administered a thymidine analog, 5-ethynyl-2′-deoxyuridine (EdU). In comparison to SPF mice, germ-free mice showed reduced uptake of EdU in both F4/80^hi^ and F4/80^lo^ phagocytes (61). This observation highlighted that commensals play a prominent role in the preservation of both the HSC-derived myeloid and splenic yolk sac–derived cells along with the inflammatory monocytes (145, 147). Further, NOD-1 is known to be responsible for mediating myeloid cell longevity. A study has shown decreased levels of NOD-1 ligand (DAP) in mice with antibiotic-altered microbiota, and upon NOD-1 stimulation, they found an abundance of IL-17–secreting lymphocytes in the intestine, which relay the microbial detection for systemic control of the phagocyte life span (148).

## Cross Talk of Gut Microbiota and ILCs

ILCs are known as the subset of innate leukocytes of lymphoid morphology that are mainly located in the mucosa. They lack antigen-specific rearranged receptors and have been grouped into NK cells and three classes as ILC1, ILC2, and ILC3 based on the type of cytokines they produce (149). Non-specific memory NK cells generated upon cytokine stimulation have been shown to exhibit a strong immune response to infections.

Amongst ILCs, group 3 ILCs have a key role in the innate immune response to invading pathogens in the gut. They are the prominent source of IL-22 and other antimicrobial proteins (AMPs) in the lamina propria of the intestine. IL-22 binds to receptors present on the intestinal epithelial cells (IECs) and induce the secretion of AMPs (RegIIIγ and RegIIIβ), which eventually limit the colonization of pathogens such as *Citrobacter rodentium* (150–152). Further, the functions of ILC3 are influenced by the gut microbial metabolites (153). Gut microbiota also aids in the conditioning and development of ILCs (154, 155). Further, germ-free and antibiotic-treated mice have a relatively diminished subset of NCR+ RORγt+ ILC population (156–158). A recent study revealed that TLR-2 agonists can directly bind to human RORγt+ ILC, inducing the secretion of IL-2, which further triggers the release of IL-22, a cytokine known to be implicated in antimicrobial defense (159). It would be interesting to see whether the interaction between gut microbiota and these ILCs imparts them the memory traits.

## IECs as a Mediator of Microbiota and Immune Cell Interaction

IECs stand as a single-cell barrier between the intestinal microbiota and the submucosal immune cells. When the IEC barrier senses microbial pathogens, it reinforces its integrity and thus protects against pathogen invasion (160). IECs expressing TLRs are stimulated by commensal-derived ligands, triggering the release of proinflammatory cytokines such as IL-6 and TNF-α (14). Further, gut-derived metabolite butyrate binds the IECs and triggers the innate sensors such as NLRP3 inflammasome to secrete IL-18 (112, 161). Commensal bacteria–derived peptidoglycan has been shown to trigger NOD-1 receptor in IECs. This event is accompanied by the production of defensin molecule and chemokine CCL20 secretion, which compel the generation of isolated lymphoid follicles (ILFs), which are the site of B cell recruitment and immune response generation (162). Moreover, there are intestinal mononuclear phagocytes (iMPs), residing in the intestinal sub-epithelium (163). Although the ontology of iMPs is still in debate, they comprise macrophages and DCs that regulate intestinal homeostasis (164). It is possible that these cells with copious expression of several PRRs upon stimulation by gut microbial components get memory signatures and persist in secondary organs.

Intestinal epithelial stem cells (IESCs) are crucial cells in the gut that have the capacity to differentiate into IECs. Gut microbiota composition has a significant impact on the IESC activity and renewal of intestinal epithelium, which is located at proximity to the lumen (165). Interestingly, SCFAs such as butyrate serve as a source of energy for IECs in metabolic processes and act to inhibit HDAC activity in IESCs (166, 167). Although it not very conclusive as of now to state the particular species of bacteria that is specifically responsible for such regulation, it is clear that some microbial metabolites stimulate the Wnt/β-catenin pathway and maintain the IESCs (168). Other signaling pathways, namely the JAK and STAT pathways, are very important in the bacteria-modulated epithelium homeostasis via stem cell regulation (165, 169, 170). These cells may serve as the potential niche for the generation of long-term innate memory.

## Future Direction and Concluding Remarks

The development of non-specific innate immune memory appears to be a crucial evolutionary phenomenon to benefit and protect the host against a variety of pathogens. Gut microbiota performs the intricate function of immune system maturation in neonates (171). Thus, the induction of memory in innate cells during this process appears to be a part of the host–microbiome co-adaptation to mediate a prompt response to the infectious stimulus.

It is evident that there would be circumstances in which the innate memory can lead to the deleterious systemic inflammatory response. If that persists for a long time, and activates innate cells in conditions such as sepsis, it can lead to tissue damage or immune paralysis. Further, the differential generation of innate memory in various organs, its duration in various immune cells, and the signaling pathways induced by gut microbial components need to be investigated in the future.

In conclusion, the process of innate memory generation should be considered as an effective approach to boost host defense and well-managed to minimize side effects while being favorable to the host. Beneficial commensals or derived products that induce the effective innate memory with regulated inflammatory response can be utilized as potential novel therapeutics to treat infections and diseases.

## Author Contributions

SNe and JA contributed to the conception of the idea, design, and conceptualization of the manuscript. SN, DD, SP, SNa, and JA contributed to the review of literature, drafting, and revision of the manuscript.

### Conflict of Interest

The authors declare that the research was conducted in the absence of any commercial or financial relationships that could be construed as a potential conflict of interest.
